# Understanding School Anxiety in Italian Adolescence through an Artificial Neural Network: Influence of Social Skills and Coping Strategies

**DOI:** 10.3390/children10101688

**Published:** 2023-10-14

**Authors:** Francisco Manuel Morales-Rodríguez, Juan Pedro Martínez-Ramón, Manuel Alejandro Narváez Peláez, Catalda Corvasce

**Affiliations:** 1 Department of Educational and Developmental Psychology, Campus of La Cartuja, Faculty of Psychology, University of Granada, 18011 Granada, Spain; fmmorales@ugr.es; 2 Department of Evolutionary and Educational Psychology, Faculty of Psychology and Speech Therapy, Campus Regional Excellence Mare Nostrum, University of Murcia, 30100 Murcia, Spain; 3 Department of Human Physiology and Physical and Sports Activity, Faculty of Medicine, University of Malaga, 29071 Málaga, Spain; mnarvaez@uma.es; 4 Liceo Statale “Carlo Cafiero”, 76121 Barletta, Italy; catalda.corvasce@liceocafiero.net

**Keywords:** coexistence, coping, school anxiety, social skills, stress

## Abstract

School anxiety depends on multiple factors that occur directly or indirectly in the teaching–learning process, such as going to the blackboard in class or reporting low grades at home. Other factors that influence school climate are social skills and coping strategies. That said, the aim of this research was to analyze the sources of school anxiety, coping strategies, and social skills in Italian secondary school students through an artificial neural network. For this purpose, a quantitative and ex post facto design was used in which the Inventory of School Anxiety (IAES), the Coping Scale for Children (EAN), and the Questionnaire for the Evaluation of Social Skills student version (EHS-A) were administered. The results showed that cognitive avoidance and behavioral avoidance coping strategies, together with the lack of social skills in students, are the variables that contributed the most to school anxiety scores in the artificial neural network. The conclusions revolve around the need to develop primary prevention programs.

## 1. Introduction

School anxiety can be defined as a negative emotional response that arises in relation to specific school situations [1,2,3]. It is characterized by feelings of fear and unease, worrisome thoughts, and a variety of physical symptoms, such as palpitations, sweating, stomachaches, headaches, and muscle tension [4,5,6]. School anxiety is a commonly observed phenomenon in the academic environment, affecting a significant proportion of the student population [7]. According to the World Health Organization, approximately 16% of the global youth population experience anxiety to some degree; this prevalence may vary depending on factors such as geographic location, cultural differences, and level of education [8]. The COVID-19 pandemic has exacerbated challenges for Italian adolescents, leading to heightened feelings of school anxiety, loneliness, academic struggles, and mental health issues [9]. This study found that 14.3% of the participants had self-harming tendencies or suicidal thoughts, emphasizing the need for support from parents, educators, and healthcare professionals. COVID-19 restrictions, including distance learning, had a profound psychological impact on Italian adolescents, particularly females, with 35% exhibiting stress reactions [10]. In addition, after the COVID-19 pandemic, the percentage increased by 25% [8]. These feelings of anxiety may be related to academic aspects (such as performance on exams or class assignments), social interactions (such as public speaking or socializing with peers), and/or institutional aspects (such as school rules and structures) [11]. Extremely high levels of anxiety have been detected in 27.3% of the Italian student body, high levels in 11.3%, and medium levels in 61.4%, using a sample of 6075 students [12]. Anxiogenic symptoms were also detected in another study conducted with Italian students [13].

Likewise, the coping strategies implemented by Italian students exert a direct impact on their levels of school anxiety [14]. Coping strategies are defined as the cognitive and behavioral efforts that individuals employ to manage internal and external demands that are perceived as exceeding their resources [15]. Coping strategies play a crucial role in the management of school anxiety [16]. These strategies can be aimed at changing the stressful situation or at regulating the feelings associated with the stress [17,18,19]. Regarding school anxiety, the existing literature suggests a significant relationship between coping strategies and anxiety levels in students [2]. Problem-focused coping strategies, such as problem-solving and seeking social support, are often associated with lower levels of school anxiety; in contrast, ineffective emotional coping strategies, such as avoidance, rumination, or self-criticism, may contribute to increased levels of anxiety [20,21,22].

On the other hand, the relationship between school anxiety and social skills has been the subject of increasing interest in recent academic research [23]. Social skills in schoolchildren refer to a set of learned behaviors that enable students to interact effectively and appropriately with peers and adults [24,25]. They are a set of behavioral and emotional competencies that enable students to interact effectively and appropriately with peers and adults [26]. These competencies include the ability to communicate clearly, listen attentively, cooperate with others, express thoughts and feelings assertively, and handle conflict situations in a respectful way [27,28,29,30]. Social skills are fundamental for the psychosocial development and academic success of students [31,32]. They are related to higher self-esteem, better interpersonal relationships, lower incidence of problem behaviors, and better academic performance [33,34]. These skills are essential for successful adaptation in social situations and may include a variety of behaviors, such as cooperation, assertiveness, empathy, and communication skills, as well as catering to the diversity of the student body [35,36]. It has been observed that students with adequate social skills tend to experience lower levels of anxiety compared to their peers with less developed social skills [37,38,39]. What is relevant about these behaviors is that such skills can be taught and improved through intervention programs at school and at home and are an essential part of social-emotional education [40].

Effective social competence can attenuate anxiety by facilitating successful interpersonal interactions, increasing self-esteem, and promoting feelings of belonging in the school environment [41]. Conversely, deficiencies in social skills may result in negative experiences, such as peer rejection or isolation, which may, in turn, intensify school anxiety [42]. Furthermore, there is evidence to suggest that anxiety can interfere with the development and manifestation of effective social skills [43]. Anxiety may hinder a student’s ability to engage in social interactions, which may limit opportunities to learn and practice social skills [44].

Likewise, the use of effective coping strategies and the possession of good social skills can help reduce anxiety levels [45,46,47]. The complexity of these relationships necessitates an integrated approach that addresses both school anxiety and social skills development in school intervention programs.

Current academic literature suggests that both coping strategies and social skills play a key role in predicting anxiety levels in students [48,49]. Coping strategies refer to the cognitive and behavioral efforts that individuals use to manage stressful situations [50,51]. It has been observed that the use of adaptive coping strategies, such as problem-solving or cognitive reappraisal, can help reduce school anxiety [52,53]. In contrast, maladaptive coping strategies, such as avoidance or rumination, may increase school anxiety [54,55,56].

In addition, the absence of effective social skills may contribute to higher levels of school anxiety [38,57]. Students who lack adequate social skills may have difficulty establishing and maintaining satisfying relationships with peers, which can result in experiences of rejection or isolation and ultimately increase school anxiety [58,59]. Therefore, a comprehensive approach that considers both coping strategies and social skills could be beneficial in predicting and managing anxiety levels in students.

In the field of statistical treatment of these variables, artificial neural networks (ANN) are advanced machine learning techniques that have been used to model and predict various human behaviors and conditions, including the variables of educational psychology [60,61,62]. ANNs are especially useful because of their ability to model nonlinear and complex relationships between predictor and outcome variables, which can be particularly relevant when studying psychological phenomena such as school anxiety [63,64]. There are recent studies that have implemented ANNs to predict anxiety levels based on a variety of predictor variables, including coping and communication strategies [60,65]. For example, a recent study used an artificial neural network to predict student anxiety based on several predictor variables in the educational domain [66]. The results of this study indicated that the artificial neural network was effective in predicting anxiety. This type of ANN-based approach could accurately predict anxiety levels and be useful to identify those students who are at risk of experiencing school anxiety. On the other hand, due to the complexity of the subject matter, there is currently a research gap based on the lack of studies that introduce artificial intelligence to predict psychological variables in the school setting and, more specifically, with specific regions and populations such as Italian high school students. Further research is needed on how coping strategies and social skills can influence school anxiety in this group, developing predictive models that help in early detection and, subsequently, more tailored action.

Having said that, the general objective of this research was to analyze the sources of school anxiety, coping strategies, and social skills in Italian secondary school students. Given the above, a number of research questions arise: (Question 1) Is there a relationship between the levels of school anxiety and the use of coping strategies and social skills in Italian high school students? (Question 2) What is the contribution of coping strategies and the lack of social skills in predicting the levels of school anxiety in these students? To answer these questions, the following specific objectives were proposed: (1) to examine the interrelation among school anxiety, coping mechanisms, and social competencies; (2) to ascertain the predictive capacity of coping mechanisms and the deficiency in social competencies in determining school anxiety levels, utilizing an artificial neural network model.

The proposed hypotheses are the following: (h1) school anxiety in Italian high school students is expected to be directly and significantly related to the use of coping strategies based on unproductive coping and a high need for increasing social skills; (h2) coping strategies and social skills (independent variables) are expected to contribute significantly to the prediction of school anxiety levels (dependent variable) in Italian high school students in a backpropagation algorithm artificial neural network model.

## 2. Materials and Methods

### 2.1. Design and Procedure

A quantitative, cross-sectional, ex post facto design study was carried out. An ex post facto design allows for identifying associations between variables but not definitive causal relationships due to its observational nature and potential confounding variables. As for the procedure, the educational center was contacted, and the management team was informed of the purpose of the study. Once their consent and that of the families of the participating students had been obtained, the questionnaires were administered. Since the original language of the questionnaires was Spanish, they were translated into Italian by a committee made up of two native Spanish researchers and a native Italian researcher with a university degree. It was translated from Spanish into Italian and back into Spanish to ensure the consistency of the original meaning by considering the International Test Commission Guidelines for test translation and adaptation [67,68]. Once the translation and verification process were completed, questionnaires were administered during a session of approximately 50 min maximum, although most of the students finished before that time, with the majority finishing between 25 and 30 min. The decision to allocate 50 min for questionnaire dissemination was influenced by the number and complexity of the items, ensuring participants had ample time to thoughtfully respond; furthermore, considering that a typical class duration can extend to 60 min, this timeframe seamlessly integrates into standard academic schedules, balancing attention span and logistical considerations. In addition, the questions were described to ensure that the students understood the instrument perfectly, which meant an increase in the application time. The administration was collected by group class and flexible groupings of between 5 and 20 students, in general, to guarantee the comprehension of the task, as well as for reasons of participation, since the participation of students whose families did not wish to participate had to be ruled out.

### 2.2. Participants

In total, a sample of *N* = 133 subjects was available of which 99 students were finally analyzed, which represented a 25.56% (n = 34) statistical mortality; 52.5% (n = 52) were girls. The students were in the fourth year of an Italian secondary school in the Bari region. Assessing school anxiety in Italian adolescents is valuable as it considers unique cultural factors, providing insights into how these young individuals experience and manage anxiety within their specific context. Participation was uninterruptedly voluntary, confidential, and anonymous. Moreover, at all stages of the research, the students were able to refuse participation without adverse consequences. As regards ethical criteria, the guidelines of the Helsinki Protocol for human research were followed. This study was approved by a university ethics committee.

### 2.3. Instruments

The psychometric tools administered were as follows:

*School Anxiety Inventory* [69,70]. This is a psychometric instrument designed in a self-report format that assesses school anxiety levels by means of 55 items and a Likert-type scale with five response options in which 0 means “never” and 4 refers to “always”. Following is an example of an item: “Being treated with contempt or with an air of superiority”. The higher the score, the higher the school anxiety. García-Fernández et al. [69] found the following Cronbach’s alpha values for each dimension according to the type of anxiety: social evaluation (α = 0.93), school failure, punishment, and aggression (α = 0.92), school evaluation (α = 0.88), cognitive and physiological (α = 0.86), and behavioral (α = 0.82). The internal consistency through Cronbach’s alpha was α = 0.76. In the present investigation, Cronbach’s alpha coefficient ranged between 0.78 and 0.89, administering its Italian translation “Inventario dell’ansia scolastica” [71]. Therefore, the “School Anxiety Inventory” was selected for this research due to its specific focus on measuring anxiety within the educational context. Its items are tailored to capture stressors and challenges students face in school settings. Additionally, its established validity and reliability ensure that the data collected is both accurate and consistent, making it a trusted instrument in the field of educational psychology. 

*Cuestionario para la Evaluación de las Habilidades Sociales-Versión para el Alumnado (A)-* [72]. This is an instrument composed of 10 items and a Likert-type scale of 10 response options, where 1 meant “never” and 5 referred to “always”, was administered. Following is an example of an item: “Sometimes I have broken or lost things that are not mine” (item 2). The higher the score, the lower the social skills, so this questionnaire should be interpreted as an instrument that assesses deficit levels in aspects related to social skills. Cronbach’s alpha coefficient in the present investigation was α = 0.79. For this study, a translation of the instrument into Italian “Questionario per la valutazione delle abilità sociale -Versione per studenti (A)- EHS-A” [72] was administered. This instrument was chosen for this study due to its comprehensive assessment of social skills specifically tailored for students. Published by the Consejería de Educación from a specific region of Spain, this questionnaire is both regionally relevant and culturally appropriate. Its design ensures that it captures the nuances of social interactions within the educational context of Spain, making it an ideal instrument for our research objectives.

*Coping Scale for Children (EAN)* [73]. This is a psychometric instrument based on a self-report that assesses coping strategies through three response options (never, sometimes, and many times) along 35 items. Following is an example of a questionnaire item: “I try to solve the problem using all possible means” (item 3). It assesses the way of dealing with four specific focuses: family, school, health, and society. The higher the score, the greater the use of the coping strategy. In total, it differentiates between nine problem-focused coping strategies: indifference (items 1, 13, 18, and 33; α = 0.52), aggressive behavior (items 7, 16, 24, and 32; α = 0.70), keeping the problem to oneself (items 9, 17, 26, and 30; α = 0.69), cognitive avoidance (items 2, 21, and 35; α = 0. 59), behavioral avoidance (items 11, 23, and 29; α = 0.41), active solution (items 3, 10, 19, and 28; α = 0.75), communicating the problem to others (items 4, 15, 22, and 31; α = 0.62), seeking information and guidance (items 5, 12, 20, and 27; α = 0.66), and positive attitude (items 8, 14, 25, and 34; α = 0.72). The first five coping strategies are grouped in a macro dimension called unproductive coping (α = 0.85), while the remaining four coping strategies are part of the macro dimension of problem-focused coping (α = 0.85). In the present investigation, the following Cronbach’s alpha coefficients were found for the first-order variables that will be subsequently used as dependent variables for the programming of the artificial neural network: indifference (α = 0.43), aggressive behavior (α = 0. 52), keeping the problem to oneself (α = 0.63), cognitive avoidance (α = 0.51), behavioral avoidance (α = 0.71), active solution (α = 0.65), communicating the problem to others (α = 0.60), information seeking and guidance (α = 0.67), and positive attitude (α = 0.78). An Italian translation was administered for this study [18]. EAN was selected for this research due to its specialized focus on understanding the coping techniques and approaches used by children. Its questions are tailored to highlight the unique methods children adopt to handle challenges. Recognized for its accuracy and consistency, the EAN offers a detailed insight into children’s resilience and adaptability, aligning perfectly with the goals of our study.

### 2.4. Data Analysis

A descriptive analysis was performed in which the main indexes of dispersion, central tendency, frequencies, and percentages were studied. An inferential analysis was also performed. To study the relationship between scale variables, Pearson’s correlation coefficient was applied. To investigate the contribution of coping strategies and social skills to the prediction of school anxiety, a multilayer perceptron artificial neural network was designed with the dichotomized variable school anxiety as the dependent variable in the output layer. This dependent variable was the result of dichotomizing a continuous variable using the 50th percentile as the cutoff point. For the design of the artificial neural network (ANN) architecture, a fixed randomness seed of 11,865,691 was set, referring anecdotally to Lévy’s constant. The initial value of lambda was 0.0000005 and the initial sigma was 0.00005. Sixty-six percent (*n* = 31) of the cases were assigned to the training phase, 21.3% (*n* = 10) to the testing phase, and 12.8% (*n* = 6) to the holdout phase. The input layer consisted of 10 units as covariates, with the order being as follows: (1) need for increasing social skills, (2) indifference, (3) aggressive behavior, (4) keeping the problem to oneself, (5) cognitive avoidance, (6) behavioral avoidance, (7) active solution, (8) communicating the problem to others, (9) seeking information and guidance, and (10) positive attitude. The method of scale shifting for the covariates was standardized. The number of units in the hidden layer was 2, and the activation function was a hyperbolic tangent. As for the output layer, school anxiety consisted of two levels: low anxiety and high anxiety, with the number of units being 2, the activation function was softmax, and the error function was cross-entropy. Finally, it is necessary to point out an extra unit of bias in the input layer and in the hidden layer. 

## 3. Results

### 3.1. School Anxiety, Need for Increasing Social Skills, and Coping Strategies 

The descriptive analysis with the main indices of dispersion and the central tendency for each of the quantitative variables that will later be analyzed through an artificial neural network is shown in Table 1. Each variable is described in terms of its average value (mean), variability (standard deviation), range (minimum to maximum values), skewness (indicating the direction and degree of asymmetry of the distribution), and kurtosis (indicating the “tailedness” of the distribution). The highest average score was observed for “School Anxiety”, indicating that this was a prominent concern among the participants. On the other hand, “Indifference” and “Cognitive Avoidance” had relatively lower average scores, suggesting that these were less prevalent or there were less intense feelings or behaviors concerning them among the group. This contrast underscores the significant anxiety levels faced by participants, while also indicating that they might not typically resort to indifference or cognitive avoidance as primary coping mechanisms. 

The study of the relationship between the continuous variables with school anxiety, as well as their confidence intervals at a significance level of 95%, are shown in Table 2. In examining the correlations with “School Anxiety”, the variable “Social Skills” exhibited the most pronounced positive correlation (r = 0.454), indicating a notable association between enhanced social skills and increased school anxiety. This suggests that students with better social skills might be more attuned to their social environments, potentially leading to heightened anxiety. Another variable, “Reserving”, also showed a significant positive correlation (r = 0.340) with school anxiety, suggesting that students who exhibit more reserved behavior might experience higher levels of anxiety in school settings. These two variables stand out in their relationship with school anxiety and warrant further exploration to understand the underlying dynamics.

### 3.2. Prediction of School Anxiety Using an Artificial Neural Network Architecture

Regarding the multilayer perceptron ANN model, in the training phase, the cross-entropy error was 19.299, the percentage of incorrect predictions was 22.6%, and the stopping rule used was after a consecutive step with no decrease in error. In relation to the testing phase, the cross-entropy error was 5.171 and the percentage of incorrect predictions was 20%. Third, in the standby phase, the percentage of incorrect predictions was 16.7%.

**Figure 1:**
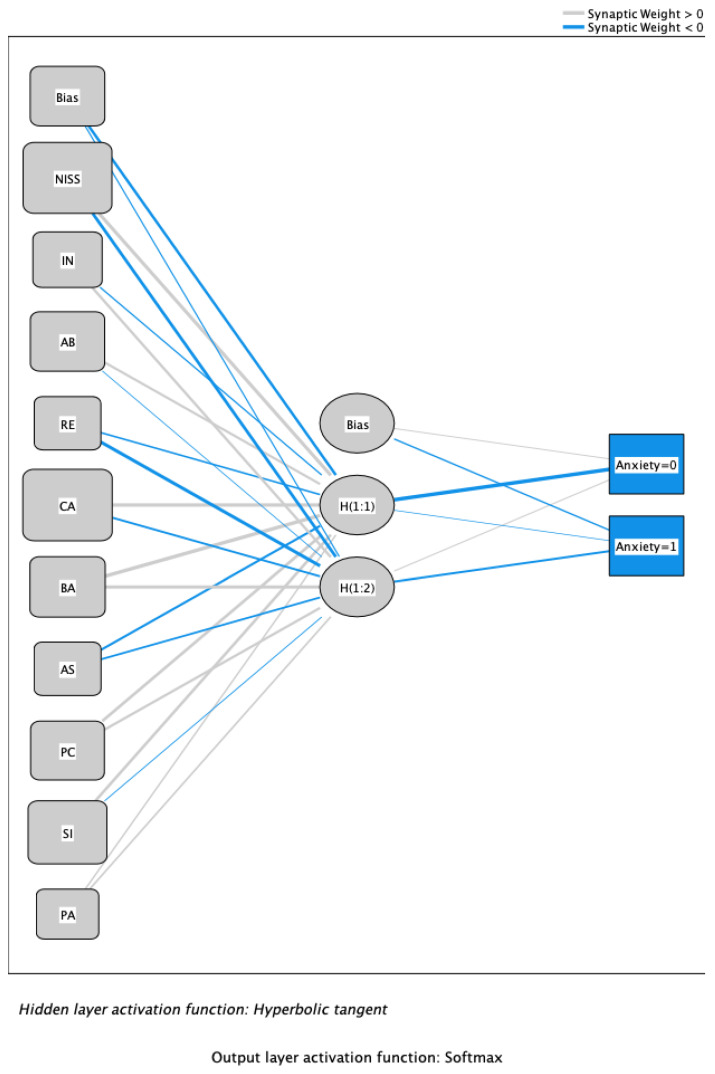
ANN for the school anxiety output variable. Note: NISS: need for increasing social skills; IN: indifference; AB: aggressive behavior; RE: reserving; CA: cognitive avoidance; BA: behavioral avoidance; AS: active solution; PC: problem communication; SI: search for information; PA: positive attitude; anxiety = 0: low school anxiety; anxiety = 1: high school anxiety. Source: own elaboration.

Figure 1 shows the ANN configuration. Synaptic weights above 0 are represented by a light line, while synaptic weights below 0 are represented by dark lines. The thickness of the line is greater the stronger the positive or negative relationship.

The parameter estimates or synaptic weights are shown in Table 3. It shows the relationships between the different nodes of the input layer with the hidden and output layers.

The predictive capacity of ANN for the dependent variable school anxiety in the output layer is shown in Table 2. The percentages of correctly predicted cases in each of the three phases are shown in Table 4. Specifically, an increase in the percentage of correctly predicted cases was found from the training phase to the testing phase and from the testing phase to the holdout phase.

**Figure 2:**
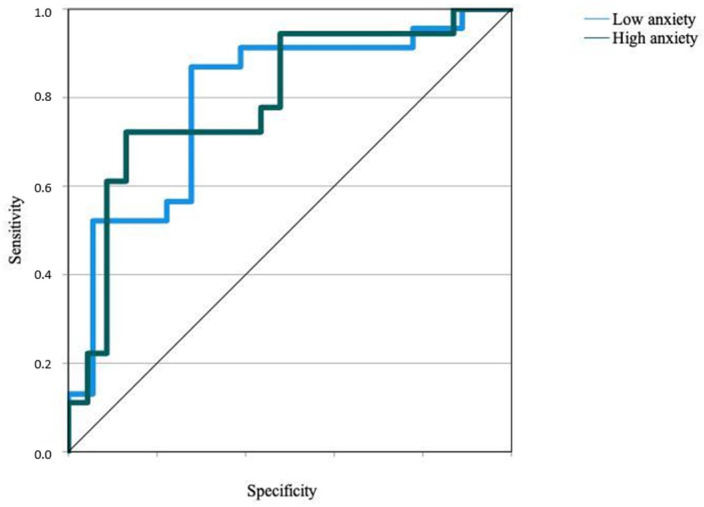
ROC curve of specificity versus sensitivity of the dependent variable school anxiety. Source: own elaboration.

Figure 2 presents the specificity versus sensitivity plot for the distribution of scores for the two output layer nodes. The area under the curve (AUC = 0.795) was greater than the random value (0.5) marked by the diagonal line dividing the quadrant, which is positive, at a 45-degree angle. This ROC (receiver operating characteristic) curve could be considered proof of a good model fit, specifically, through the discriminative capacity of the model for the dichotomous variable.

The contribution of each variable or node of the input layer to the predictive capacity of the ANN for the dependent variable school anxiety of the output layer is shown in Table 5 by its importance (index ranging from 0 to 1) and normalized importance (from 0 to 100). The data present the importance and standardized importance of various behavioral and cognitive factors as determined by an artificial neural network (ANN) model. Notably, “Cognitive Avoidance” emerged as the most influential factor, boasting the highest standardized importance of 100.0%. Closely following was the “Need for Increasing Social Skills” with a significant standardized importance of 98.1%. On the other hand, “Positive Attitude” appeared to exert the least influence on the model, which is reflected by its minimal standardized importance of 9.5%. Other factors, such as “Aggressive Behavior” and “Search for Information and Guidance”, held moderate influence with standardized importance values around the 50% mark.

## 4. Discussion

The purpose of this research was to investigate the roots of anxiety in the school context, coping techniques, and social skills among high school students in Italy. As for hypothesis 1, according to which it was anticipated that, among Italian high school students, academic anxiety levels would have a direct and remarkable relationship with the adoption of coping techniques focusing on ineffective coping and those with a marked lack of social skills, this hypothesis is confirmed. Our findings corroborate and extend the current understanding of school anxiety. The definition of school anxiety provided by Reid [1] aligns with what was observed in our research. This issue has also been addressed globally by the World Health Organization [10] and specifically with the Italian population [12]. It is crucial to note that our observations on the coping strategies of Italian students and their relationships with school anxiety are supported by previous studies [14]. We agree with Lazarus and Folkman [15] that coping strategies can be considered as responses to stress beyond individual capabilities. However, it is critical to discern between adaptive and maladaptive coping strategies. Our findings support the idea that problem-focused strategies tend to decrease anxiety, whereas maladaptive ones may intensify it [20]. Furthermore, our research found a direct relationship between poor social skills and school anxiety, something that has been the subject of interest in recent studies [28,38,41,43]. Students with insufficient social skills tend to experience more anxiety, which is consistent with previous research [37,39]. This connection highlights the importance of boosting the development of social skills, as they have been shown to be essential for academic success and psychosocial development [31]. Deficiencies in social skills can lead to negative experiences such as peer rejection, further enhancing school anxiety [42]. Our study supports the idea that effective social competence reduces anxiety by enhancing interpersonal interaction and increasing self-esteem, which is in line with previous studies [41]. Likewise, the present study also found a relationship between school anxiety and keeping the problem to oneself, which can be explained if we consider that students who do not ask for help feel more agitated because they cannot share it and do not expect to receive help. On the other hand, paradoxically, actively seeking a solution and communicating the problem are strategies that were associated with higher levels of school anxiety. It may be that students interpret these strategies as stressful situations that require the use of resources that cause them anxiety. The findings of this research provide a comprehensive understanding of the intricate relationship between school anxiety, coping techniques, and social skills among Italian high school students. The paradoxical association of active problem-solving and communication with increased school anxiety warrants a more in-depth exploration, especially when juxtaposed against the backdrop of Lazarus and Folkman’s theory [15] on coping strategies. A question needs to be raised: are Italian students uniquely predisposed to perceive these strategies as stress-inducing, or is this a broader phenomenon observed across cultures? More research needs to be conducted.

As for hypothesis 2, according to which it was expected that, in an artificial neural network model using the backpropagation algorithm, both coping strategies and social skills (independent variables) would have a significant impact on the estimation of academic anxiety levels (dependent variable) in high school students in Italy, this hypothesis is, finally, confirmed. In the present investigation, a predictive capacity of 83.3% was achieved as well as an area under the curve greater than 0.5, and it is, therefore, above randomization. These results are in line with others that demonstrate that artificial neural networks have the potential to be a valuable tool for predicting variables in educational psychology [60,61,62]. In the field of the study of school anxiety, this variable has also been analyzed with positive results through neural networks [66]. As for which variables provide the most information to understand their contribution to anxiety, in the present study, the factors that most influenced the predictive capacity were the cognitive avoidance coping strategy and the lack of social skills or the need to increase them. These data are in line with the idea that social skills and coping strategies are closely related to school anxiety as previously observed [48,49]. From the present research, it was found that students with a lack of social skills and who use cognitive avoidance as a coping strategy are more likely to suffer from anxiety. It is thus demonstrated that dysfunctional or maladaptive strategies produce or are associated with higher levels of anxiety, which is in line with other authors [54]. In summary, school anxiety is a considerable challenge faced by many students, but with appropriate interventions focused on coping strategies and social skills, students can be offered essential tools to manage and reduce their anxiety levels. Regarding the model obtained and based on an ANN, when comparing the cross-entropy errors and incorrect predictions with prior studies in educational psychology, the similarity in the results bolsters the validity of our model, suggesting that it is consistent with previous research and is well-calibrated, notwithstanding that there remains room for optimization [60,61,62]. Within the domain of educational psychology, traditional statistical methodologies, such as regression and ANOVA, have long been the gold standard for data analysis, primarily due to their linear nature and foundational insights. However, the emergence of artificial neural networks (ANNs) has introduced a dynamic shift in this landscape. ANNs, inspired by biological neural structures, excel in capturing intricate, non-linear relationships inherent in human behaviors and cognitive processes, which are often overlooked by conventional methods. Their ability to efficiently handle vast datasets and high-dimensional data positions them as a potent tool for modern educational research, especially with the proliferation of data from online educational platforms. Thus, while ANNs offer a complementary perspective, it is imperative to view them within the broader context of statistical methodologies in educational psychology, ensuring a balanced and comprehensive approach to research [60,61,62,63].

### 4.1. Applicability

There are several intervention programs and educational practices focused on teaching and improving these skills, which are recognized as a critical component of social-emotional education [74]. This research may contribute to the understanding of school anxiety in Italian high school students and may be useful in developing intervention programs or educational policies to address this problem based on the updated data on the current situation provided through this study. In this sense, the knowledge of school anxiety levels may provide valuable information to guide prevention and treatment interventions. In addition, these findings hold valuable insights for educational practitioners and policymakers. For instance, recognizing the pivotal role of social skills in school anxiety emphasizes the need to integrate social skills development programs into the curriculum. Similarly, the observed relationship between coping strategies and anxiety suggests the importance of incorporating stress management and the training of coping skills in educational settings. Furthermore, understanding the impact of social competence in reducing anxiety underscores the significance of fostering positive peer relationships and inclusive school environments. Lastly, acknowledging the role of communication in anxiety highlights the importance of open channels for students to express their concerns. A more comprehensive exploration of these pedagogical implications can guide evidence-based interventions that prioritize students’ mental well-being and create a supportive educational atmosphere.

### 4.2. Limitations

One of the limitations of this study was the small number of participants due to the difficulties in accessing the sample. In addition, the high amount of data lost by the system in several variables, especially regarding school anxiety, should be considered as another limitation of the study. Statistical mortality, or the loss of participants during the study, could indeed influence the validity and generalizability of the results. Given this rate of attrition, there is a possibility that the participants who remained might differ in some systematic way from those who dropped out, potentially introducing bias. It is necessary to interpret the results with caution as the generalizability of the data may be affected. In this sense, special attention should be paid to minimizing the number of subjects who do not answer the questionnaires, for example, by changing the order in which the instruments are administered. By identifying gaps in current understandings and how the results challenge or expand existing theories, a deeper comprehension of the studied phenomena could be achieved. Likewise, carrying out a study with different countries entails the need to learn and understand the specific procedures required to reach the educational centers and their classrooms, explain the object of the study, obtain the appropriate consent, and administer the questionnaires. Therefore, this research should be considered as a preliminary study of school anxiety and the contribution of coping strategies and lack of social skills in Italian students.

### 4.3. Further Lines of Research

This research can be the starting point for the development of comparative studies that analyze cultural expectations, social norms, or parenting styles that may influence students’ anxiety levels and the strategies they use to manage it. Another future line of research that is in line with the limitations of the present study is to increase the number of participants to improve the generalizability of the results to other contexts. Finally, given the impact that climate change is having on the psychological conditions of the population, it would be interesting to include the analysis of eco-anxiety in the educational community [75]. While this study primarily focuses on school anxiety among adolescents, the mention of eco-anxiety is justified as it acknowledges the broader sociocultural context in which adolescents navigate their anxieties. Increasingly, concerns about environmental issues and climate change have become integral parts of young people’s lives, intersecting with their educational experiences. Recognizing eco-anxiety in tandem with school anxiety highlights the multifaceted nature of adolescents’ emotional well-being, acknowledging that external stressors, including environmental concerns, can impact their overall mental health and educational experiences. This point along with social competences could be a further line of research [76].

## 5. Conclusions

School anxiety is problematic among Italian students. Cognitive avoidance and lack of social skills are the variables that contribute most to school anxiety scores in the artificial neural network. On the other hand, there is a clear correlation between certain coping strategies and school anxiety. A direct and significant relationship was found between the coping strategy of reserving—keeping the problem to oneself—and higher school anxiety. There is also a direct and significant correlation between this and the lack of social skills as would be expected. There is no doubt that social skills are fundamental to the psychosocial well-being of students. The lack or deficit of certain skills can amplify school anxiety, while effective social competence can reduce these anxiety levels by promoting healthier and more successful interactions. However, the fact that some coping strategies initially considered to be adaptive have been directly correlated with school anxiety levels implies the need for further research on this topic. Although our research and the current literature provide an approximation to understanding the relationship between school anxiety, coping strategies, and social skills, it is essential to continue with this line of study. Social and academic dynamics are fluid and may change over time, so it is crucial to keep up with current trends and challenges.

Eventually, we reiterate the importance of a comprehensive approach to school intervention programs that address school anxiety and encourage the development of effective social skills and coping strategies. The inherent complexity of these relationships demands careful attention and proactive action to improve student well-being.

## Figures and Tables

**Table 1:** Descriptive analysis of school anxiety; need for increased social skills and coping strategies.

	*M*	*SD*	Min.	Max.	Skewness	Kurtosis
School anxiety	157.45	98.56	8	428	0.60	−0.08
Need for increasing social skills	21.51	7.00	10	42	0.58	0.23
Indifference	2.84	1.52	0	6	−0.22	−0.22
Aggressive behavior	2.87	1.70	0	7	0.11	−0.42
Reserving	3.91	2.12	0	8	−0.05	−0.60
Cognitive avoidance	2.85	1.57	0	6	0.09	−0.33
Behavioral avoidance	4.03	2.15	0	8	−0.00	−0.40
Active solution	4.75	1.97	0	8	−0.10	−0.65
Problem communication	4.28	1.97	0	8	0.04	−0.21
Search for information	4.28	1.94	0	8	−0.03	−0.21
Positive attitude	5.11	2.09	0	8	−0.27	−0.72

Note. M: media; SD: standard deviation; Min: minimum; Max: maximum. Source: own elaboration.

**Table 2:** Pearson correlation of the variable “school anxiety” with the rest of the continuous variables and confidence intervals.

Variables Correlated with “School Anxiety”	Pearson Correlation	Sig. (2-Tailed)	95% C. I. ^(1)^	
			Lower	Upper
Social skills	0.454	<0.001	0.202	0.644
Indifference	0.139	0.307	−0.130	0.387
Aggressive Behavior	0.191	0.154	−0.075	0.429
Reserving	0.340	0.011	0.079	0.553
Cognitive avoidance	0.177	0.193	−0.092	0.419
Behavioral avoidance	0.196	0.149	−0.073	0.435
Active solution	0.307	0.020	0.048	0.524
Problem communication	0.284	0.034	0.021	0.507
Search for information	0.109	0.419	−0.157	0.359
Positive attitude	−0.022	0.872	−0.283	0.242

Note. ^(1)^ Estimation is based on Fisher’s r-to-z transformation with bias adjustment.; C. I.: confidence intervals. Source: own elaboration.

**Table 3:** ANN parameter estimates.

Predictor		Predicted			
		Hidden Layer 1		Output Layer	
		H (1:1)	H (1:2)	[Anxiety = 0]	[Anxiety = 1]
Input layer	(Bias)	−0.285	−0.067		
	Need for increasing social skills	0.483	−0.400		
	Indifference	−0.069	0.256		
	Aggressive behavior	0.250	−0.049		
	Reserving	−0.123	−0.438		
	Cognitive avoidance	0.530	−0.198		
	Behavioral avoidance	0.537	0.421		
	Active solution	−0.240	−0.176		
	Problem communication	0.393	0.255		
	Search for information	0.391	−0.054		
	Positive attitude	0.122	0.168		
Hidden layer 1	(Bias)			0.058	−0.122
	H (1:1)			−0.681	−0.039
	H (1:2)			0.066	−0.236

Note: anxiety = 0: low school anxiety; anxiety = 1: high school anxiety. Source: own elaboration.

**Table 4:** Percentage correctly predicted for each of the ANN stages.

Stage	Observed	Predicted		
		0	Low Anxiety	Percent Correct
Training	Low anxiety	14	1	93.3%
	High anxiety	6	10	62.5%
	Overall Percent	64.5%	35.5%	77.4%
Testing	Low anxiety	7	1	87.5%
	High anxiety	1	1	50.0%
	Overall Percent	80.0%	20.0%	80.0%
Holdout	Low anxiety	3	0	100.0%
	High anxiety	1	2	66.7%
	Overall Percent	66.7%	33.3%	83.3%

Note. Dependent variable: school anxiety (low vs. high). Source: own elaboration.

**Table 5:** Importance of ANN independent variables.

	Importance	Standardized Importance
Need for increasing social skills	0.195	98.1%
Indifference	0.065	32.7%
Aggressive behavior	0.098	49.4%
Reserving (or keeping the problem to oneself)	0.047	23.7%
Cognitive avoidance	0.199	100.0%
Behavioral avoidance	0.105	52.6%
Active solution	0.049	24.6%
Communicating the problem to others	0.094	47.4%
Search for information and guidance	0.129	64.7%
Positive attitude	0.019	9.5%

Source: own elaboration.

## Data Availability

The data can be requested by the scientific community in the ethical terms to be determined.
